# Preclinical Characterization of the Radioimmunoconjugate ^111^In or ^90^Y-FF-21101 Against a P-Cadherin–Expressing Tumor in a Mouse Xenograft Model and a Nonhuman Primate

**DOI:** 10.2967/jnumed.120.245837

**Published:** 2021-02

**Authors:** Yuichi Funase, Eri Nakamura, Masamichi Kajita, Yasutaka Saito, Shinobu Oshikiri, Michi Kitano, Masahiko Tokura, Akihiro Hino, Tomoya Uehara

**Affiliations:** 1 RI Research Department, Fujifilm Toyama Chemical Co., Ltd., Chiba, Japan; 2 Graduate School of Pharmaceutical Sciences, Chiba University, Chiba, Japan; and; 3 Project Management Department, Fujifilm Toyama Chemical Co., Ltd., Tokyo, Japan

**Keywords:** targeted radionuclide therapy, theranostics, CDH3, antibody, dosimetry

## Abstract

P-cadherin is overexpressed in various cancers and can be a target for radioimmunotherapy. We investigated the preclinical pharmacokinetics and pharmacology of FF-21101, an ^111^In- or ^90^Y-conjugated monoclonal antibody against P-cadherin, to evaluate its clinical applications. **Methods:** The radiochemical purity, binding affinity, and in vitro serum stability of ^111^In or ^90^Y-labeled FF-21101 were evaluated. The pharmacokinetics of ^111^In or ^90^Y-FF-21101 were compared in normal mice. Tumor accumulation after ^111^In-FF-21101 administration was investigated in mice bearing subcutaneous tumors with high (NCI-H1373), moderate (EBC-1), or no (A549) P-cadherin expression. The tumor suppression effect after a single intravenous injection of ^90^Y-FF-21101 was assessed in NCI-H1373 and EBC-1 mouse xenograft models. The relationship between antibody dose and tumor accumulation was investigated in the NCI-H1373 mouse xenograft model. The absorbed radiation dose in humans after injection of ^90^Y-FF-21101 was estimated using γ-camera images of cynomolgus monkeys. **Results:** The radiochemical purities of ^111^In- and ^90^Y-FF-21101 were 98.2% ± 2.5% (*n* = 9) and 99.3% ± 0.6% (*n* = 5), respectively. The dissociation constants were 1.083 nM for ^111^In-FF-21101 and 1.367 nM for ^90^Y-FF-21101. Both ^111^In- and ^90^Y-FF-21101 were stable in human serum after 96 h of incubation and exhibited similar pharmacokinetics in normal mice. The tumor accumulation of ^111^In-FF-21101 was closely related to the intensity of P-cadherin expression in the cells. ^90^Y-FF-21101 showed significant tumor growth inhibition, indicating that NCI-H1373 and EBC-1 recurrence was not observed after intravenous administration of 3.7 and 7.4 MBq, respectively of ^90^Y-FF-21101 per animal. Tumor uptake in the mouse xenograft model and estimated absorbed radiation doses in the spleen of monkeys decreased with increasing antibody doses of ^111^In-FF-21101. Conversely, the estimated absorbed radiation dose in the red marrow increased with increasing antibody dose. An antibody dose of 4.8 mg/m^2^ was considered appropriate for humans, on the basis of efficacy and safety. The maximum tolerated administered activity of ^90^Y-FF-21101 was estimated to be 2,886 MBq/human. **Conclusion:** FF-21101 radioimmunotherapy exhibited high antitumor affinity and antitumor efficacy in mouse xenograft models. Extrapolation of the pharmacokinetics in monkeys to humans suggests the potential for clinical application of FF-21101 for treating P-cadherin–expressing tumor.

## Visual Abstract

**Figure:**
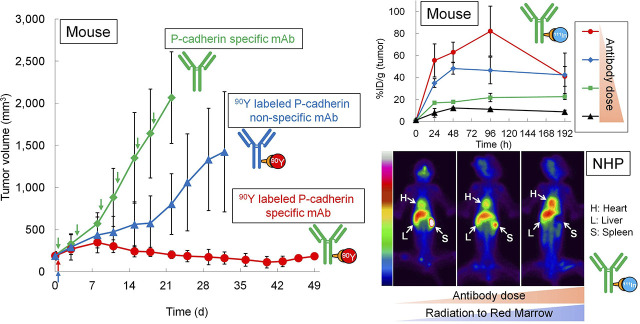

The cell-to-cell adhesion molecule known as P-cadherin is overexpressed in several tumors, including breast, colon, lung, and pancreas (1–3), and is implicated in tumor cell motility, migration, and invasiveness (4). P-cadherin is also involved in epithelial–mesenchymal transition (5), cancer stem cell mediation (6), and breast cancer susceptibility gene mutations (7) and is associated with poor prognosis in various cancers (2,8–11). Conversely, P-cadherin expression levels are low in normal tissues (3). Therefore, P-cadherin is considered to be an attractive target for solid-tumor treatment. PF-03732010 is a humanized anti–P-cadherin monoclonal antibody that exhibited high therapeutic efficacy in preclinical studies (12). However, PF-03732010 failed to exert therapeutic efficacy in the phase 1 trial (NCT00557505) (13), suggesting insufficient pharmacologic activity for solid tumor treatment.

To enhance the pharmacologic activity, we investigated ^90^Y-conjugated anti–P-cadherin monoclonal antibody, considering the successful therapeutic efficacy of ^90^Y-ibritumomab tiuxetan, a ^90^Y-labeled anti-CD20 antibody, against non-Hodgkin lymphoma (14). Since P-cadherin expression is high in tumors and low in normal tissues, it can facilitate a biodistribution appropriate for achieving high efficacy with radioimmunotherapy. The phase 1 trial of PF-03732010 showed that a weekly dosage of 15 mg/kg of body weight was well tolerated, suggesting that the unpredictable toxicity of anti–P-cadherin antibodies is unlikely.

FF-21101, a 1,4,7,10-tetraazacyclododecane-1,4,7,10-tetraacetic acid (DOTA)–conjugated chimeric human/mouse monoclonal antibody of IgG_1_ (PPMX2032), targets human P-cadherin. PPMX2032 has no anticell proliferation activity. DOTA forms inert radiometal chelates with ^111^In and ^90^Y (15,16). The β-particles emitted from ^90^Y induce cellular damage in target and neighboring cells via high-energy β-radiation and free radicals (17). ^90^Y-FF-21101 constitutes an armed antibody strategy for solid tumor treatment and may be more effective than anti–P-cadherin therapy alone.

This study assessed the efficacy and safety of combining P-cadherin–targeting and radionuclide therapy. We evaluated the tumor-suppressive effects of ^90^Y-FF-21101, FF-21101, and ^90^Y-labeled P-cadherin nonspecific antibody in a mouse xenograft model and estimated the clinical safety of ^90^Y-FF-21101 in humans by evaluating biodistribution in nonhuman primates and then extrapolating those results to patients.

## MATERIALS AND METHODS

### Antibodies

PPMX2032 and FF-21101 were obtained from Perseus Proteomics Inc. and Fujifilm Diosynth Biotechnologies U.S.A. Inc.

Whole-molecule human IgG (hIgG) and *S*-2-(4-isothiocyanatobenzyl)-1,4,7,10-tetraazacyclododecane tetraacetic acid (SCN-Bn-DOTA) were purchased from Thermo Fisher Scientific Inc. and Macrocyclics Inc. hIgG was incubated with 50 mM bicine and 150 mM sodium chloride buffer (pH 8.5) containing SCN-Bn-DOTA for 17 h at 25°C. Purification of DOTA-conjugated hIgG (DOTA-hIgG) and buffer exchange with 250 mM sodium acetate (pH 5.5) were performed using PD-10 desalting columns (GE Healthcare).

### Radiolabeling

FF-21101 and DOTA-hIgG were labeled with ^111^In (^111^InCl_3_) (Nordion) or ^90^Y (^90^YCl_3_) (CIS Bio International). The antibodies were incubated with ^111^InCl_3_ or ^90^YCl_3_ in 250 mM sodium acetate (pH 5.5) for 15 min at 45°C and purified with phosphate-buffered saline (PBS, pH 7.4) using NAP-5 columns (GE Healthcare). The radiochemical purities (>90%) were analyzed using Tec-Control chromatography strips 150-771 (Biodex) developed with saline containing 10 mM diethylenetriaminepentaacetic acid. Nonlabeled antibodies or PBS (pH 7.4) was used to adjust the protein concentration.

### Binding Assay

The dissociation constants (Kds) of ^111^In-FF-21101 and ^90^Y-FF-21101 were evaluated with a saturation binding assay. sCDH3C (ECD1–ECD2 of human P-cadherin-mouse Fc fusion protein), an antigen of FF-21101, was obtained from Perseus Proteomics Inc. Eight-well strip plates, clear corner–notched (Thermo Fisher Scientific), were treated with 250 μL of 1% glutaraldehyde solution per well at 37°C for 2 h. The plates were incubated with a of 20 μg/mL concentration of sCDH3 (100 μL/well) dissolved in 100 mM sodium acetate buffer at 25°C for 2 h, with shaking and subsequent overnight incubation at 25°C. To determine nonspecific binding, 100 mM sodium acetate buffer was added to the negative control wells. The plates were treated with 100 mM lysine sodium phosphate (150 μL/well) at 37°C for 1 h, followed by incubation with 10 mM calcium chloride (200 μL/well) containing 1% Block Ace (KAC Co.) at 37°C for 2 h with shaking. Radiolabeled FF-21101 (10 mg/mL) was diluted in 8 steps of 2-fold serial dilution with 10 mM calcium chloride containing 0.4% Block Ace. Each sample (100 μL/well) was added to the wells and incubated at 37°C for 3 h. Ten millimolar of calcium chloride containing 0.1% Block ace was used as the washing buffer in each step. Radioactivity in each well and 100 μL of each serial dilution as a total count were measured with a γ-counter. Kd and maximum binding values were calculated via nonlinear regression analysis using Prism software (version 5.04; GraphPad Software) (1-site binding analysis equation: *y* = maximum binding × *x*/[Kd + *x*]).

### In Vitro Stability in Serum

^111^In-FF-21101 and ^90^Y-FF-21101 were added to human serum (Access Biologicals) at a concentration of 0.5 MBq/5 μg/mL (*n* = 3). The methodology is described in Supplemental Method 1 (supplemental materials are available at http://jnm.snmjournals.org) (18–20). The serum samples were analyzed after incubation for 0, 3, 24, 48, and 96 h at 37°C using sodium dodecyl sulfate poly-acrylamide gel electrophoresis. The mixture of each sample and 100 mM diethylenetriaminepentaacetic acid at 9:1 was mixed with an equal amount of Novex Tris-glycine sodium dodecyl sulphate sample buffer (Thermo Fisher Scientific) and heated at 85°C for 2 min. Electrophoresis was performed using Novex 4%–20% Tris-glycine mini gels and Novex Tris-glycine sodium dodecyl sulphate running buffer (Thermo Fisher Scientific). The electrophoresed gels were exposed to Storage Phosphor Screen BAS IP (GE Healthcare) at −20°C, and radioactivity was measured using Typhoon FLA 7000IP (GE Healthcare); the resulting images were analyzed using Multi Gauge (FUJIFILM, Tokyo, Japan). Data were normalized to 100% at t = 0.

### Animal Studies

All animal studies were performed in accordance with the Institutional Animal Care and Use Committee of Fujifilm Toyama Chemical Co., Ltd., and adhered to the Guidelines for Proper Conduct of Animal Experiments issued by the Science Council of Japan (June 1, 2006).

### Comparative Biodistribution of ^111^In-FF21101 and ^90^Y-FF-21101 in Normal Mice

Eight-week-old BALB/c mice (Charles River Laboratories Japan) were stratified on the basis of body weight and randomly assigned to 10 groups (5 mice per group) using the statistical analysis software Exsus (CAC Croit Corp.). The mice were intravenously administered 0.5 MBq/40 μg ^111^In-FF-21101 (5 groups) or ^90^Y-FF-21101 (5 groups). At 5 min and 24, 48, 96, and 192 h after administration, the blood, heart, lungs, liver, kidneys, spleen, and bones were removed and weighed. Nonbone tissues were dissolved in SOLVABLE (PerkinElmer) overnight at 50°C under shaking conditions; bones were dissolved in hydrochloric acid overnight at 37°C under shaking conditions. The dissolved tissue fluids and counting standards of ^111^In and ^90^Y were examined for radioactivity; the tissue radioactivity level, expressed as percentage of administered activity remaining in a tissue (%ID/g), was calculated from the weights and radioactivity levels of the collected tissues. Pharmacokinetic parameters were estimated from each time point as mean %ID/mL of blood using the WinNonlin pharmacokinetic software (Certara) with the noncompartmental approach.

### Cell Culture

The human lung adenocarcinoma cell lines NCI-H1373 and A549 were purchased from the American Type Culture Collection. The human lung squamous cell carcinoma cell line EBC-1 was purchased from the Japanese Collection of Research Bioresources Cell Bank. NCI-H1373, EBC-1, and A549 were selected as cell lines with high, moderate, and no P-cadherin expression, respectively (21). The following cell culture media, containing 10% fetal bovine serum (Moregate Biotech) and 1% penicillin/streptomycin (Thermo Fisher Scientific), were used: minimum essential medium (Thermo Fisher Scientific) for EBC-1, Roswell Park Memorial Institute 1640 medium (Thermo Fisher Scientific) for NCI-H1373, and Ham F-12K (Kaighn) medium (Thermo Fisher Scientific) for A549. The cultures were maintained at 37°C under a humidified atmosphere (5% CO_2_).

### Mouse Xenograft Model

Male BALB/c Slc-*nu/nu* mice (7–8 wk old; Japan SLC) were subcutaneously inoculated with 5 × 10^6^ cells/0.1 mL of NCI-H1373, EBC-1, or A549 suspended in Dulbecco PBS in the right flank. One to 2 wk after transplantation (until the mean tumor volume reached approximately 200 mm^3^), the mice were randomized on the basis of tumor volume and body weight to different groups, using the statistical analysis software Exsus. The tumor volume was calculated as [long diameter × (short diameter)^2^]/2.

### Biodistribution in Mouse Xenograft Models

^111^In-FF-21101 or ^111^In-labeled DOTA-hIgG (^111^In-hIgG) (740 kBq/30 μg) was administered to the NCI-H1373, EBC-1, and A549 mouse xenograft models via the tail vein. The mice were euthanized by exsanguination under anesthesia at 5 min and at 24, 48, 96, and 192 h after administration (*n* = 3/time point). All organs of interest (blood, brain, heart, lungs, liver, spleen, pancreas, stomach, small intestine, cecum, large intestine, kidneys, adrenals, adipose tissue, testes, bone, muscle, skin, thyroid, and tumor) were removed. The tissue weights and the radioactivity were measured.

### In Vivo Efficacy

We estimated the therapeutic efficacy of ^90^Y-FF-21101 as follows. On day 0, the NCI-H1373 and EBC-1 mouse xenograft models were assigned to the following 4 groups (*n* = 6/group): single administration of PBS, 10 mg/kg administration of PPMX2032 twice a week for 3 wk, single 3.7 MBq/30 μg administration of ^90^Y-FF-21101, and single 3.7 MBq/30 μg administration of ^90^Y-labeled DOTA-hIgG (^90^Y-hIgG).

A radiation dose-escalation study was also performed. On day 0, the EBC-1 mouse xenograft model was assigned to the following 3 groups (*n* = 6/group): single administration of PBS, single 3.7 MBq/30 μg administration of ^90^Y-FF-21101, and single 7.4 MBq/30 μg administration of ^90^Y-FF-21101.

The injectates were administered from day 1 to each mouse via the tail vein. The tumor volume and the body weight were measured twice a week. The mice were euthanized when their body weight decreased by more than 20% from that at day 0 or if the tumor volume exceeded 10% of the total body weight. The time point at which individual mice were euthanized was defined as the endpoint of the group.

### Antibody Dose Optimization in Mouse Xenograft Model

^111^In-FF-21101 was administered at 740 kBq/3.73, 30, 112, or 373 μg to NCI-H1373-inoculated mice via the tail vein. The mice were euthanized by exsanguination under anesthesia at 5 min and at 24, 48, 96, and 192 h after administration (*n* = 3/time point), and the weight and radioactivity of the extirpated tumor were measured.

### Biodistribution and Dosimetry in Cynomolgus Monkeys

This study was conducted on 3 adult cynomolgus monkeys (Hamri Co.), after bolus intravenous administration of ^111^In-FF-21101. The monkeys were administered approximately a 37 MBq/kg dose of ^111^In-FF-21101 at an antibody dose of 0.04, 0.4, or 4 mg/kg. Blood samples were collected by venipuncture at 0.17, 0.5, 1, 3, 6, 24, 48, 72, 96 (or 120), 144, 168, and 216 h after the administration. The radioactivity of the blood samples and the counting standards (diluted administered solution) was measured using a γ-counter to calculate %ID/mL of blood.

$$\text{\%ID/mL of blood}=\frac{\text{radioactivity of blood }\left(\text{cpm/mL}\right)}{\text{administered activity }\left(\text{cpm}\right)}\times 100.$$

$$\text{Administered activity }\left(\text{cpm}\right)=\text{counting standard activity }\left(\text{cpm}\right)\times \text{dilution factor}\times \frac{\text{ administered volume }\left(\text{mL}\right)}{\text{counting standard volume }\left(\text{mL}\right)}.$$

On the basis of the %ID/mL values of the blood, the biologic pharmacokinetic parameters were estimated using the WinNonlin pharmacokinetic software with the noncompartmental approach, which is consistent with the intravenous bolus route of administration. All parameters were generated from the scaled %ID/mL of blood:

$$\text{Scaled \%ID/mL}=\frac{\text{\%ID}}{\text{max \%ID}}\times 100,$$

where max %ID = maximum value of %ID × monkey blood volume (mL), monkey blood volume (mL) = monkey body weight (kg), and monkey circulating blood volume = 65 mL/kg (22).

Each monkey was administered general anesthesia and underwent whole-body planar scintigraphy with a calibration source (diluted administered solution) at 3, 6, 24, 48, 72, 96 (or 120), 144, and 168 h after administration using Symbia E (Siemens). The radioactivity of the calibration source was measured using a γ-counter to calculate ratio of calibration source.

$$\text{Ratio of calibration source}=\frac{\text{calibration source activity }\left(\text{cpm}\right)}{\text{administered activity }\left(\text{cpm}\right)}.$$

The acquisition parameters were set as follows. The matrix was 256 × 512, the scan speed was 5 cm/min (until 48 h after administration) or 3 cm/min (after 72 h after administration), and the energy window was set to 15% for both 173 and 247 keV. The scanner was configured with a medium-energy low-penetration collimator for the data acquisition. A region of interest (ROI) was set for the detectable organs; each ROI count was calculated using the Syngo MI Application VA60C (Siemens). Tissue %ID was calculated from each ROI count. The ROI of the background was set to be within the 4 corners of the field of view in each of the anterior and the posterior images. The ROI of the body background was set at 8 locations in muscle in each of the anterior and posterior whole-body images. The count per pixel (CPP) for the background and the body background were calculated for each of the anterior and posterior images. The ROIs of the calibration source, whole body, heart, liver, spleen, and lungs were set along the shapes of the accumulations. The counts of the calibration source, the whole body, and each organ were calculated in each of the anterior and posterior images, where count of calibration source = ROI count of calibration source − CPP of background × ROI size of calibration source, count of whole body = ROI count of whole body − CPP of background × ROI size of whole body, and count of organ = ROI count of organ − CPP of body background × ROI size of organ. The counts for each ROI in the anterior and posterior images were averaged and used for calculation of %ID:

$$\text{\%ID of whole body}=\frac{\text{averaged count of whole body}}{\text{administered count}}\times 100.$$

$$\text{\%ID of organ}=\frac{\text{averaged count of organ}}{\text{administered count}}\times 100.$$

$$\text{Administered count}=\frac{\text{averaged count of calibration source}}{\text{ratio of calibration source}}.$$

The time-integrated activity coefficients of red marrow were calculated from blood radioactivity. The %ID/mL of blood at each time point was converted to the fraction of injected activity per mL (FIA/mL). FIA/mL values were performed for decay correction and humanization, and scaled such that the maximum value of FIA (max FIA) would be 1.0. These calculations are shown in below. A 64 h half-life was used for ^90^Y.

$$\text{Scaled FIA/mL}=\frac{\text{humanized FIA/mL}}{\text{humanized max FIA}},$$

$$\text{Humanized FIA/mL }=\text{decay}-\text{corrected FIA/mL}\times \frac{\text{monkey body mass}}{\text{reference body mass}},$$

where reference body mass (male) = 73,700 g (default value of OLINDA/EXM 1.0), humanized max FIA = maximum value of humanized FIA/mL × reference blood volume, reference blood volume (male) = 5,300 mL (19), decay=corrected FIA/mL = FIA/mL × 0.5^(t/half-life)^, and FIA/mL = %ID/mL / 100.

The time-integrated activity coefficients of the blood (T_blood/mL_) were obtained using noncompartmental analysis of scaled FIA/mL values with WinNonlin. The time-integrated activity coefficients of the red marrow (T_redmarrow_) were calculated according to the following formula (23):

$${\text{T}}_{\text{redmarrow}}={\text{T}}_{\text{blood}/\text{mL}}\times \text{reference red marrow mass}\times \frac{\text{RMECFF}}{1-\text{HCT}},$$

where reference red marrow mass (male) = 1,120 g (default value of OLINDA/EXM 1.0), RMECFF (red marrow extracellular fluid fraction) = 0.19 (24), and HCT (hematocrit) = 0.47.

We calculated the time-integrated activity coefficients for the monkey from planar imaging by plotting and integrating the measured effective activities for each imaged source organ over some number of time points. The %ID for each ROI was converted to the FIA (%ID/100). The time-integrated activity coefficients of the organ were obtained using noncompartmental analysis of the FIA values with WinNonlin. The time-integrated activity coefficients of the remainder were calculated by subtracting the time-integrated activity coefficients of other tissues from that of the whole body. The absorbed dose estimates were computed using OLINDA/EXM 1.0 (Hermes Medical Solutions) from the time-integrated activity coefficients of each organ. Source organs were red marrow, heart contents, liver, lungs, spleen, and remainder of body. The nuclide ^90^Y and the adult male reference model were selected for the input form of OLINDA/EXM.

### Statistical Analysis

Statistical analyses were performed using the statistical analysis software Exsus. Significance was determined using the Steel–Dwass test at day 22 (NCI-H1373) and day 18 (EBC-1) for comparing the tumor volume between the PBS, PPMX2032, ^90^Y-hIgG (3.7 MBq/animal), and ^90^Y-FF-21101 (3.7 MBq/animal) administration groups. *P* values of less than 0.05 were considered statistically significant. Data are presented as mean ± SD.

## RESULTS

### Radiolabeling, Binding Activity, and Serum Stability In Vitro

**FIGURE 1.**
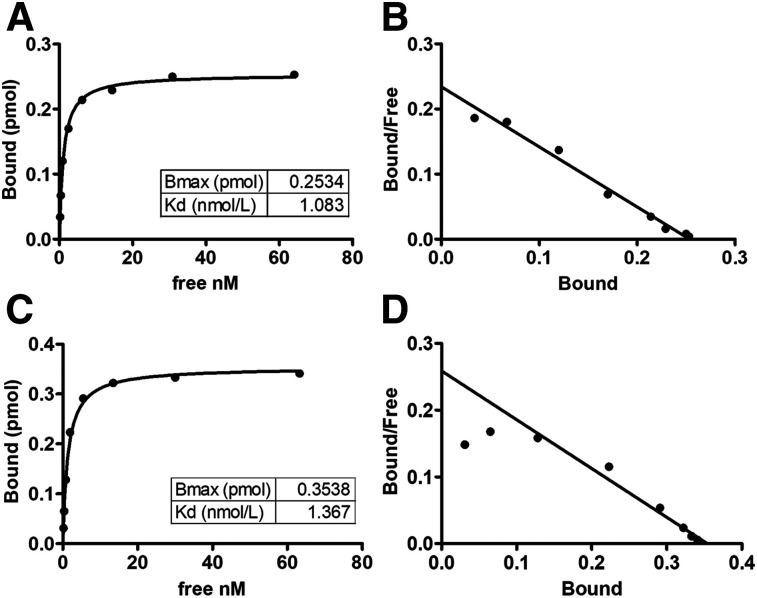
Binding activity of radiolabeled FF-21101 to sCDH3C: saturation binding curve of ^111^In-FF-21101 (A), Scatchard plot of ^111^In-FF-21101 (B), saturation binding curve of ^90^Y-FF-21101 (C), and Scatchard plot of ^90^Y-FF-21101 (D). *Bound* refers to amount of radiolabeled FF-21101 bound to sCDH3C. *Free* refers to concentration of free radiolabeled FF-21101. Bmax = maximum binding.

The radiochemical purities (mean ± SD) of ^111^In-FF-21101, ^90^Y-FF-21101, ^111^In-hIgG, and ^90^Y-hIgG were 98.2% ± 2.5% (range, 91.6%–99.9%; *n* = 9), 99.3% ± 0.6% (range, 98.2%–99.7%; *n* = 5), 99.4% ± 0.3% (range, 99.2–99.6; *n* = 2), and 99.2% ± 0.6% (range, 98.7%–99.6%; *n* = 2), respectively. The Kds of ^111^In-FF-21101 and ^90^Y-FF-21101 were 1.083 and 1.367 nM, respectively (Fig. 1). Both ^111^In-FF-21101 and ^90^Y-FF-21101 remained stable in serum for 96 h (Table 1).

**TABLE 1:** Stability of Radiolabeled FF-21101 in Human Serum

Agent	Incubation time			
	3 h	24 h	48 h	96 h
^90^Y-FF-21101	96.9 ± 0.2	94.7 ± 0.3	94.3 ± 0.4	93.7 ± 0.7
^111^In-FF-21101	99.8 ± 0.2	99.3 ± 1.5	99.3 ± 0.2	98.1 ± 1.5

Values are presented as mean ± SD (*n* = 3) for relative change (%) of intact.

### Comparative Pharmacokinetics of ^111^In or ^90^Y-FF-21101 in Normal Mice

**FIGURE 2.**
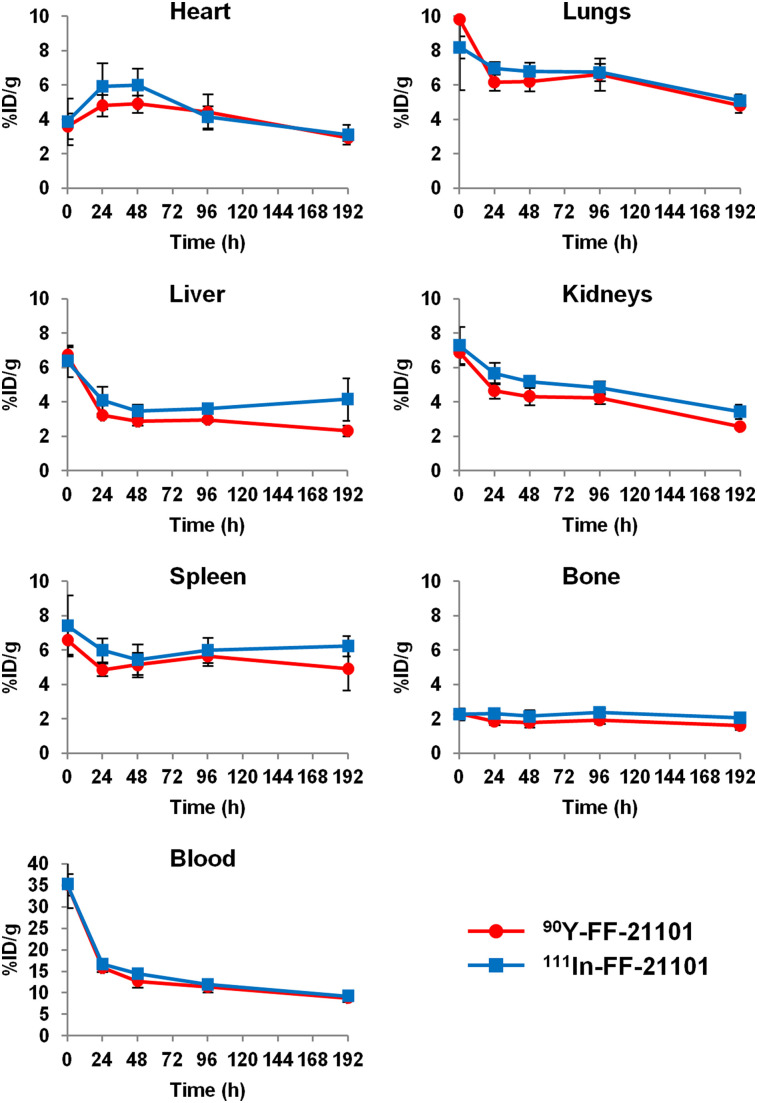
Time–activity (%ID/g) curves for ^90^Y-FF-21101 and ^111^In-FF-21101 in each organ of normal mice. Error bars represent SD (*n* = 3 per time point).

^90^Y-FF-21101 and ^111^In-FF-21101 displayed similar pharmacokinetics. The radioactivity of ^90^Y-FF-21101 and ^111^In-FF-21101 in the blood was highest 5 min after administration (35.3 and 35.9 %ID/g, respectively). Both ^90^Y-FF-21101 and ^111^In-FF-21101 exhibited similar pharmacokinetic parameters (Table 2) and similar %ID/g versus time for each organ (Fig. 2).

**TABLE 2:** Pharmacokinetic Parameters of ^90^Y-FF-21101 and ^111^In-FF-21101 in Normal Mice

Agent	C_max_ (%ID/g)	AUC (%ID × h/g)	T_1/2_ (h)	CL (g/h)	V_ss_ (g)
^90^Y-FF-21101	35.32	5971.67	273.38	0.02	6.25
^111^In-FF-21101	35.94	5733.08	231.14	0.02	5.52

C_max_ = maximum concentration; AUC = area under the curve; T_1/2_ = half-life; CL = clearance; V_ss_ = volume of distribution at steady state.

### Antigen-Specific Tumor Accumulation

**FIGURE 3.**
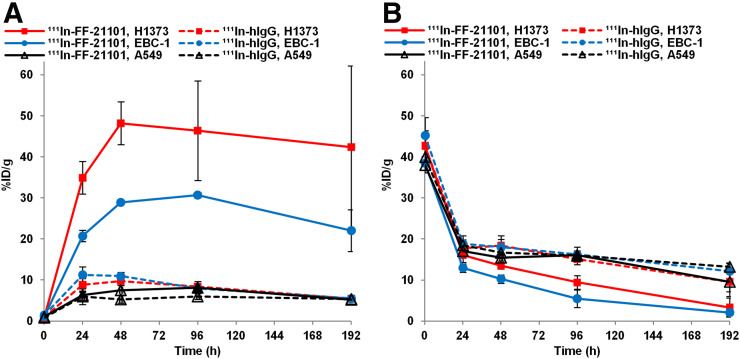
Time–activity (%ID/g) curves for uptake of ^111^In-FF-21101 and ^111^In-hIgG in tumor (A) and blood (B) in high (H1373), moderate (EBC-1), or negative (A549) P-cadherin–expressing models. Error bars represent SD (*n* = 3 per time point).

^111^In-FF-21101 showed antigen-specific tumor accumulation (Fig. 3A). The maximum ^111^In-FF-21101 tumor uptakes were 48.2 and 30.7 %ID/g in the NCI-H1373 and EBC-1 mouse xenograft models, respectively. Conversely, ^111^In-hIgG tumor accumulation was insignificant; the maximum tumor uptakes were 9.7 and 11.3 %ID/g in the NCI-H1373 and EBC-1 mouse xenograft models, respectively. ^111^In-FF-21101 and ^111^In-hIgG tumor accumulation in the A549 mouse xenograft model was also insignificant, with maximum tumor uptakes of 8.1 and 6.0 %ID/g, respectively. When tumor accumulation was low, blood radioactivity was high (Fig. 3B). P-cadherin–specific and –nonspecific antibody accumulations in other tissues were insignificant, and there were no major differences between the models (Supplemental Tables 1–6).

### Tumor Suppressive Effects

**FIGURE 4.**
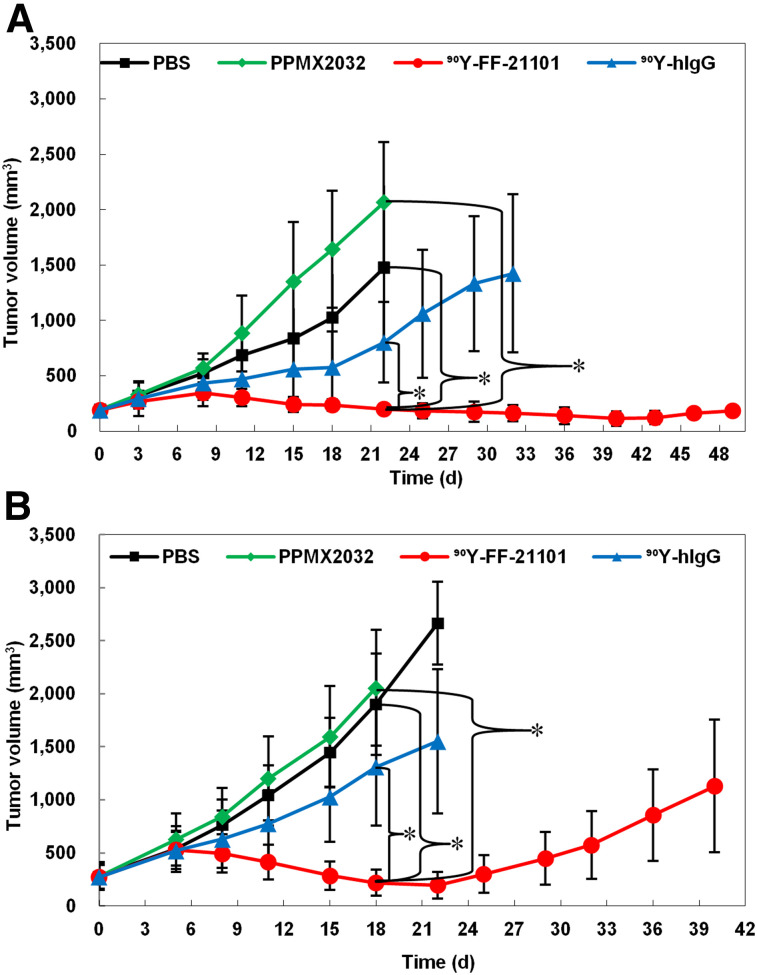
Tumor suppression in NCI-H1373 (A) and EBC-1 (B) mouse xenograft models. Tumor growth curves show mean tumor volumes ± SD (*n* = 6). Curves are plotted until first tumor per group reached 10% of total body weight. *P < 0.05.

**FIGURE 5.**
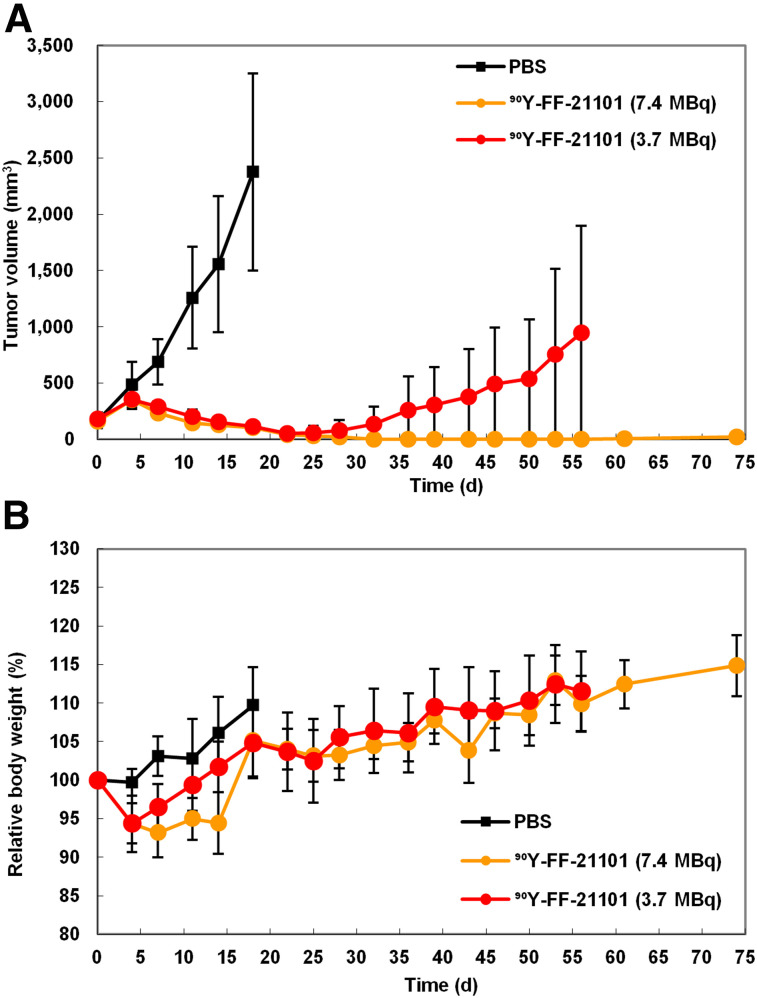
Radiation dose dependency of ^90^Y-FF-21101 in EBC1 mouse xenograft model. (A) Tumor growth curve. (B) Relative body weight. Curves show mean ± SD (*n* = 6) and are plotted until first tumor per group reached 10% of total body weight.

The mean tumor volumes at day 0 in the NCI-H1373 and EBC-1 mouse xenograft models were 191 and 277 mm^3^, respectively. No animals lost more than 20% of their body weight. The body weight did not differ between groups (Supplemental Figs. 1 and 2). ^90^Y-FF-21101 exhibited the strongest tumor suppressive effect in the 2 xenograft models (Figs. 4A and 4B). Conversely, PPMX2032 and ^90^Y-hIgG did not show significant differences from PBS in either model. Although ^90^Y-FF-21101 (3.7 MBq/animal) showed a high antitumor effect, tumor regrowth was observed in the EBC-1 model from day 22. Therefore, a radiation dose-escalation therapeutic study was conducted on the EBC-1 mouse xenograft model. The mean tumor volume at day 0 was 197 mm^3^, and tumor regrowth was not observed in the ^90^Y-FF-21101 (7.4 MBq/animal) group until day 74 (Fig. 5A). Complete tumor regression was observed in 5 mice in the ^90^Y-FF-21101 (7.4 MBq/animal) group. Although transient weight loss was observed in the ^90^Y-FF-21101 (7.4 MBq) group on days 5–15, no animals lost more than 20% of its body weight until day 74 (Fig. 5B).

### Effect of Antibody Dose on Tumor Accumulation in Mouse Xenograft Model, Pharmacokinetics in Cynomolgus Monkeys, and Estimated Absorbed Radiation Dose in Humans

**FIGURE 6.**
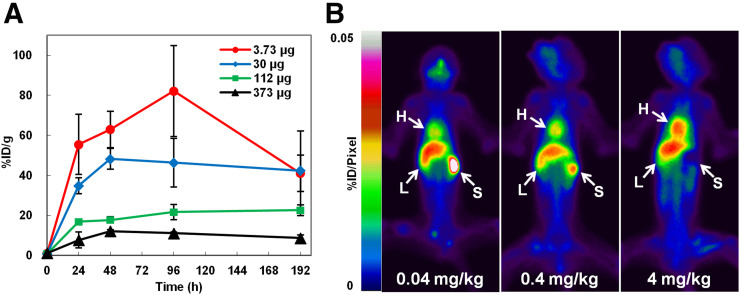
Effect of antibody dose on tumor accumulation and biodistribution. (A) Tumor accumulation for different antibody doses (3.73, 30, 112, and 373 μg/animal) of ^111^In-FF-21101 in NCI-H1373 mouse xenograft model. Results are presented as %ID/g of tumor at each time point. Error bars represent SD (*n* = 3 per time point). (B) Whole-body planar imaging of cynomolgus monkeys 48 h after ^111^In-FF-21101 administration. Locations of heart (H), liver (L), and spleen (S) are indicated by arrows. Full-field-of-view images are shown in Supplemental Figures 3–5.

Tumor uptake of ^111^In-FF-21101 in NCI-H1373–inoculated mice decreased with increases in antibody dose, and the trend persisted at each time point (Fig. 6A).

In cynomolgus monkeys, approximately 70%–80% of the administered activity of ^111^In-FF-21101 was found throughout the body 3 h after injection; radioactivity counts decreased to approximately 50%–60% at 168 h after injection. The accumulation of ^111^In-FF-21101 in the heart, liver, spleen, and lungs was 7–9, 10–12, 1–5, and 4–6 %ID at 3 h after injection, respectively. The radioactivity in the heart and lungs decreased to less than 40% at 168 h after infusion, with a slightly longer retention of radioactivity in the spleen and liver at 168 h after infusion. Radioactivity decreased in the spleen and increased in the heart with increasing antibody dose (Fig. 6B). Blood radioactivity peaked at 10 min after injection of ^111^In-FF-21101. The area under the curve and biologic half-life (T_1/2_) increased and clearance decreased with increasing antibody dose. There were no remarkable trends in the maximum concentration and volume of distribution at steady state (V_ss_) among the antibody dose levels (Table 3).

**TABLE 3:** Pharmacokinetic Parameters Calculated from Blood Radioactivity in ^111^In-FF-21101 Administered to Cynomolgus Monkeys

Parameter	Antibody dose		
	0.04 mg/kg	0.4 mg/kg	4 mg/kg
C_max_ (%ID/mL)	0.52	0.45	0.47
AUC (%ID × h/mL)	38.75	43.26	69.32
T_1/2_ (h)	147.73	147.10	241.91
CL (mL/h/kg)	0.88	0.68	0.44
V_ss_ (mL/kg)	146.93	124.61	136.14

C_max_ = maximum concentration; AUC = area under the curve; T_1/2_ = half-life; CL = clearance; V_ss_ = volume of distribution at steady state

The estimated absorbed radiation doses of ^90^Y-FF-21101 in humans were extrapolated from the results of monkeys (Table 4). Increased antibody dose significantly lowered the estimated absorbed radiation dose of ^90^Y-FF-21101 in the spleen. The estimated absorbed radiation dose slightly increased with increased antibody dose in the heart wall, red marrow, osteogenic cells, and whole body.

**TABLE 4:** Estimated Absorbed Radiation Dose (mGy/MBq) of ^90^Y-FF-21101 in Humans

Organ	Antibody dose		
	0.04 mg/kg	0.4 mg/kg	4 mg/kg
Heart wall	2.31	2.54	3.28
Liver	2.56	2.47	2.55
Lungs	1.21	1.19	1.82
Red marrow	0.661	0.786	0.885
Osteogenic cells	0.680	0.817	0.912
Spleen	12.0	6.93	2.77
Total body	0.382	0.423	0.459

## DISCUSSION

In addition to PF-03732010, tumor treatments targeting P-cadherin have been challenged for PF-06671008 (bispecific antibody against P-cadherin and CD3) (25) and PCA-062 (DM1 conjugated antibody) (NCT02375958). We chose a targeted radionuclide therapy approach using β-emitters based on the success of ^90^Y-ibritumomab tiuxetan (14) and ^177^Lu-DOTATATE (26). For application of ^90^Y-FF-21101 to clinical studies, we showed here that ^90^Y-FF-21101 could expose tumors to radioactivity required for treatment without causing serious radiotoxicity to normal tissues.

^111^In- and ^90^Y-FF-21101 showed robust radiolabeling, remained stable in human serum, and exhibited similar P-cadherin binding affinities and biodistribution; thus, ^111^In-FF-21101 could be used to estimate ^90^Y-FF-21101 pharmacokinetics.

^111^In-FF-21101 and ^111^In-hIgG were administered to mouse xenograft models with different levels of P-cadherin expression (high: NCI-H1373, moderate: EBC-1, none: A549) (21); we found that ^111^In-FF-21101 accumulation in tumors was dependent on P-cadherin expression levels. ^111^In-FF-21101 tumor accumulation in A549 was unrelated to antigen specificity because it was as low as ^111^In-hIgG tumor accumulation. Furthermore, ^111^In-hIgG accumulation in P-cadherin–positive cells was as low as that in P-cadherin–negative cells and lower than that in the blood (Figs. 3A and 3B). Therefore, differences in ^111^In-FF-21101 accumulation in tumors with different P-cadherin expression levels supported the P-cadherin–specific accumulation of ^111^In-FF-21101.

The biodistribution profiles of ^111^In-FF-21101 were well reflected in the suppressed tumor growth of the P-cadherin–positive xenografted tumor cells after ^90^Y-FF-21101 administration. The single injection of 3.7 MBq of ^90^Y-FF-21101 significantly suppressed tumor growth, compared with the groups treated with PBS, PPMX2032, or ^90^Y-hIgG. The difference in the tumor suppression period between the 2 models reflected a difference in tumor accumulation based on P-cadherin expression levels. Tumor regrowth was not observed up to day 74 in the EBC-1 mouse xenograft model that was administered 7.4 MBq of ^90^Y-FF-21101 per animal. Therefore, it was predicted that radioactivity equivalent to at least 7.4 MBq/mouse would be required in humans to obtain a high antitumor effect in clinical trials.

The effect of antibody dose on ^111^In-FF-21101 biodistribution was investigated to estimate the clinical dose. Tumor uptake of ^111^In-FF-21101 in a mouse xenograft model decreased with an increasing antibody dose, indicating that the FF-21101–binding sites on the tumor cells become saturated at high antibody doses. FF-21101 has no cross-reactivity with mouse P-cadherin; we studied pharmacokinetics and dosimetry using cynomolgus monkeys with reactive antigens for FF-21101. Furthermore, serum soluble P-cadherin levels in cynomolgus monkeys are similar to those in healthy humans and cancer patients (27). The absorbed radiation dose increased in organs affected by blood flow, such as the red marrow and heart, because of prolonged radioactivity retention in the blood with antibody dose increases. The absorbed radiation dose to the spleen decreased with an increase in antibody dose (Table 4). A similar phenomenon was observed in ^111^In-ibritumomab tiuxetan with preadministration of unlabeled anti-CD20 antibodies in humans (28). Thus, the antibody dose optimization should consider both tumor accumulation and normal-tissue distribution.

In view of the results from monkeys and mice, we estimated the clinical antibody doses of ^90^Y-FF-21101. Because the antibody dose of FF-21101 in clinical studies will be based on body surface area, the antibody doses (mg/kg) for the cynomolgus monkey were converted to dose based on body surface area (mg/m^2^). The dose was converted to dose per individual (μg/animal) for mice (Table 5; Supplemental Method 2) (29). The upper limits of the absorbed radiation dose were set at 3,000 and 20,000 mGy for the red marrow and any other organ, respectively (30), and the dose-limiting organ and maximum radiation dose were estimated at each antibody dose (Table 6). A radiation dose of 7.4 MBq/animal in the mouse xenograft model corresponded to 888 MBq/m^2^ and 1,678 MBq/human (Supplemental Method 3) (18,19,29). An antibody dose of 0.48 mg/m^2^ (1,667 MBq/human as maximum radiation dose) would result in an inadequate margin of safety for a therapeutic radiation dose of 1,678 MBq/human. The dose-limiting organ at 48 mg/m^2^ was red marrow, linked to a high radiotoxicity risk. Furthermore, 48 mg/m^2^ (equivalent to 400 μg/animal in mice) was higher than 378 μg/animal in mice as an antibody dose, suggesting that tumor accumulation was insufficient (Fig. 6A). On the basis of our calculations, an antibody dose of 4.8 mg/m^2^ for ^90^Y-FF-21101 was estimated as appropriate for humans. Because the 4.8 mg/m^2^ dose (0.13 mg/kg in humans; Supplemental Method 4 (29)) was much lower than the dose of PF-03732010 (15 mg/kg), for which toxicity was not observed in the phase 1 trial (13), the risk of toxicity derived from antibodies would also be low. Moreover, the 4.8 mg/m^2^ dose enables administration of up to 2,886 MBq/human, which is markedly higher than the estimated therapeutic radiation dose (1,678 MBq/human).

**TABLE 5:** Antibody Dose in Cynomolgus Monkey (mg/kg) and Mouse (μg/animal [25 g]) Corresponding to Antibody Dose per Body Surface Area (mg/m^2^)

Antibody dose unit (dose per each stab)	Antibody dose		
Body weight of monkey (mg/kg)	0.04	0.4	4
Body surface area (mg/m^2^)	0.48	4.8	48
Individual mouse (μg/animal)	4	40	400

**TABLE 6:** Estimated Dose-Limiting Organ and Maximum Radiation Dose at Each Antibody Dose

Antibody dose (mg/m^2^)	Dose-limiting organ	Maximum radiation dose (MBq/human)
0.48	Spleen	1,667
4.8	Spleen	2,886
48	Red marrow	3,390

Maximum radiation dose calculation is shown in Supplemental Method 5 (30).

## CONCLUSION

^111^In-FF-21101 showed P-cadherin–specific high tumor accumulation, and ^90^Y-FF-21101 significantly suppressed P-cadherin–expressing tumors. Analysis of the estimated absorbed radiation dose of ^90^Y-FF-21101 in humans predicted the antibody and radiation dose in clinical cases and showed sufficient safety. Therefore, promising outcomes can be expected for a clinical trial with FF-21101. It is reasonable to estimate the absorbed radiation dose of ^90^Y-FF-21101 using ^111^In-FF-21101 because of the similarity in biodistributions and pharmacokinetics demonstrated between the two in vivo. On the basis of these findings, a phase 1 trial in the United States (NCT02454010) was conducted using an antibody dose from 0.625 to 3.125 mg/m^2^. The expansion phase is being performed with an antibody dose of 3.125 mg/m^2^.

## DISCLOSURE

Yuichi Funase, Eri Nakamura, Masamichi Kajita, Yasutaka Saito, Shinobu Oshikiri, Michi Kitano, Masahiko Tokura, and Akihiro Hino are employees of Fujifilm Toyama Chemical Co., Ltd. No other potential conflict of interest relevant to this article was reported.

**KEY POINTS**

**QUESTION:** Is the clinical application of P-cadherin radioimmunotherapy promising?
**PERTINENT FINDINGS:** P-cadherin radioimmunotherapy demonstrated a significantly higher antitumor effect than anti–P-cadherin antibody monotherapy and P-cadherin–nonspecific radioimmunotherapy in a mouse xenograft model. On the basis of the absorbed radiation dose analysis, ^90^Y-FF-21101 was estimated to be capable of administering radioactivity sufficiently high to meet the needs for human treatment.
**IMPLICATIONS FOR PATIENT CARE:** Combining ^90^Y with P-cadherin–specific antibody may be a possible therapeutic strategy for P-cadherin–expressing cancer.
